# Liquid and Vapour Phase of Lavandin (*Lavandula × intermedia*) Essential Oil: Chemical Composition and Antimicrobial Activity

**DOI:** 10.3390/molecules24152701

**Published:** 2019-07-25

**Authors:** Stefania Garzoli, Giovanni Turchetti, Pierluigi Giacomello, Antonio Tiezzi, Valentina Laghezza Masci, Elisa Ovidi

**Affiliations:** 1 Department of Drug Chemistry and Technology, Sapienza University, 00185 Rome, Italy; 2 Department for the Innovation in Biological, Agrofood and Forestal Systems, Tuscia University, 01100 Viterbo, Italy

**Keywords:** lavandin essential oil, liquid phase, vapour phase, HS-GC/MS analysis, antibacterial activity

## Abstract

Essential oils from *Lavandula* genus and the obtained hybrids are widely used for different purposes such as perfume production in the cosmetic field and for its biological properties. This is the first study on the liquid and vapour phase of *Lavandula × intermedia* “Grosso” essential oil grown in the Lazio Region, Italy, investigated using headspace coupled to gas chromatography and mass spectrometry (HS-GC/MS). The results showed the most abundant components were linalool and linalyl acetate, followed by 1,8-cineole and terpinen-4-ol, while lavandulyl acetate and borneol were identified as minor compounds, maintaining the same proportion in both the liquid and vapour phase. Furthermore, we tested lavandin liquid and vapour phase essential oil on gram-negative bacteria (*Escherichia coli, Acinetobacter bohemicus,* and *Pseudomonas fluorescens*) and gram-positive bacteria (*Bacillus cereus* and *Kocuria marina*).

## 1. Introduction

The genus *Lavandula* belongs to the Lamiaceae family, a medicinal aromatic plant family whose essential oils (EOs) are widely used for applications in food, perfumery, and in the field of cosmetics; it is also important for human health given its potential biological activities [1,2,3,4,5,6].

*Lavandula* species are found worldwide and consist of over 39 species, besides various intraspecific taxa and hybrids, which differ in colour and period of flowering, foliage, and resistance to low temperatures [7]. *Lavandula angustifolia* (also called *L. officinalis* or *L. vera*)*, Lavandula latifolia* (spike lavender), and *Lavandula* × *intermedia* Emeric ex Loisel (lavandin or lavandulil), a natural sterile hybrid obtained by crossing *L. angustifolia* × *L. latifolia* are mainly used to produce EOs.

*L. × intermedia* is a shrub ranging from 60 to 150 cm, with linear-lanceolate to spathulate leaves, which are often tomentose. Its inflorescence stalk is branched and flowers show a corolla with a bilateral symmetry and are variable in colour from lilac-purple to white, blooming from late June to July.

Lavandin is a much larger plant than the *L. angustifolia* varieties and is much appreciated for its EO yield. This product is widely used all over the world by the fragrance industry and is a common ingredient in soaps, laundry detergents, skin care, perfumes, and cleaning products.

*L. × intermedia* is present in Spain, France, and Italy with different cultivars among which is *L. × intermedia* var. “Grosso”; it is characterised by dark violet-blue flowers and is the most widely used for EO production [7].

EOs, particularly abundant in aromatic plants, play a fundamental role in plant protection against pathogens such as bacteria, fungi, and insects. Furthermore, they attract some insects to allow the fertilization process by pollen and seed dispersion [8,9]. They are a hydrophobic complex mixture of volatile compounds and are mainly composed of terpenes biogenerated by the mevalonate pathway. These volatile molecules include monoterpenes (hydrocarbon and oxygenated monoterpens), and sesquiterpenes (hydrocarbon and oxygenated sesquiterpens) [10]. They also contain phenolic compounds derived from the shikimate pathway. Thanks to their chemical composition, EOs possess antioxidant, anti-inflammatory, antimicrobial, antifungal, and anticancer properties [11,12,13].

Different biological activities were found in common lavender and lavandin EOs such as anxiolytic, antioxidant, anti-inflammatory, antidepressant, analgesic, smooth-muscle relaxant, anti-parasitic, anticancer, and apoptosis induction activity, as well as having beneficial immunomodulatory effects on wound healing [14,15,16,17,18,19,20,21]. Moreover, it was reported that the EOs obtained from *Lavandula* are active on many types of bacteria such as foodborne pathogens, human pathogenic bacteria, and environmental bacteria [22,23,24,25,26,27]. Furthermore, *Lavandula × intermedia* and *Lavandula Angustifolia* essential oils, rich in specific constituents such as linalool, camphor, and 1,8-cineole, possess antibacterial activities against *Listeria monocytogenes,* especially against isolates from a clinical environment [28].

In the present study, we have analysed the chemical composition of the liquid and vapour phase of *L. × intermedia* cv. “Grosso” essential oil (LEO) grown in the Lazio Region, Italy, investigated by headspace coupled to gas chromatography and mass spectrometry (HS-GC/MS). We further studied LEO’s antimicrobial properties against gram-negative bacteria (G^−^) (*Escherichia coli, Acinetobacter bohemicus,* and *Pseudomonas fluorescens*) and gram-positive bacteria (G^+^) (*Bacillus cereus* and *Kocuria marina*).

## 2. Results

### 2.1. LEO Chemical Composition

GC–MS analysis of the liquid phase showed the presence of 26 compounds (Table 1), of which 89.2% were monoterpenoids and 3.1% were sesquiterpenoids The most abundant components were linalool (41.6%) and linalyl acetate (23.0%), followed by 1,8-cineole (5.2%) and terpinen-4-ol (4.8%). Lavandulyl acetate (3.2%) and borneol (2.8%) were identified as minor compounds. Headspace GC–MS analysis revealed different results. The percentages of the most volatile compounds increased while that of the highest boiling compounds decreased as shown in Table 1. All the lower boiling compounds showed a greater abundance. More specifically, α-pinene increased to 8.7% and 1,8-cineole reached 19.8%. In contrast, compounds eluted subsequently from the chromatographic column recorded a decrease in their abundance compared to the analysis of the liquid phase. For example, linalool decreased to 35.8%, linalyl acetate to 7.5%, and terpinen-4-ol to 2.9%.

Table 2 shows the qualitative aspects of LEO. The amounts of the components found were in line with the levels accepted by the European Pharmacopeia (EPh). Only linalyl acetate was slightly below the limits set by the EPh.

### 2.2. LEO Antibacterial Activity—Agar Diffusion Method

The antibacterial activity of LEO was assessed according to the inhibition zone diameter (halo) and the results obtained are summarised in Table 3. In the G^−^ group, an inhibition halo of 8.5 ± 0.7 mm was measured for *P. fluorescens*, while relative antibiotic control using gentamicin (gen) was 25.5 ± 0.7 mm; *E. coli* showed an inhibition halo of 13 ± 1.41 mm (gen = 16.5 ± 2.12 mm) whereas *A. bohemicus* exhibited a halo of 47 ± 4.24 mm (gen = 34.5 ± 0.7 mm). In the G^+^ group tested, the *K. marina* inhibition halo was 14.5 ± 2.12 mm (gen = 27 ± 2.83 mm) and that for *B. cereus* was 21.5 ± 0.7 mm (gen = 28.5 ± 0.7 mm).

### 2.3. Minimum Inhibitory Concentration (MIC)

The MIC represents the lowest concentration of antimicrobial agent that completely inhibits growth. Values are reported in percentage of LEO (Table 4) revealing that the most resistant strain was *P. fluorescens* (MIC_LEO_ = 3.75%, MIC_GEN_ = 1.56 µg/mL), followed by *E. coli* (MIC_LEO_ = 1.87%, MIC_GEN_ = 3.12 µg/mL) and *K. marina* (MIC_LEO_ = 1.87%, MIC_GEN_ = 0.39 µg/mL), *B. cereus* (MIC_LEO_ = 0.94%, MIC_GEN_ = 1.56 µg/mL), and *A. bohemicus* (MIC_LEO_ = 0.47%, MIC_GEN_ = 0.08 µg/mL). No bacterial growth occurred in the sterility control, while regular growth was recorded in the negative control. In all strains tested, DMSO antibacterial activity was not present for the MIC_LEO_ concentrations used.

### 2.4. Minimum Bactericidal Concentration (MBC)

MBC is expressed as the lowest concentration of essential oil where no visible growth on agar occurred. LEO tested on the G^+^ group displayed a bacteriostatic effect, while on the G^−^ group it recorded results that were consistent with the MIC data recorded before: *P. fluorescens* showed an MBC of 7.50%, *E. coli* was 1.87%, and *A. bohemicus* was 0.47%; higher values of both MIC and MBC were obtained by using gen as a positive control (Table 4).

### 2.5. Vapour Phase Test (VPT)

The VPT used to evaluate the antimicrobial activity of the LEO vapour phase is shown in Table 5. After 24 h of incubation time, differing results emerged: For *P. fluorescens*, the MIC was higher than 40 µL whereas *K. marina*, *E. coli*, and *B. cereus* recorded an MIC of 20 µL. *A. bohemicus* recorded the lowest level, with an MIC of 2 µL. After seven days the plates were checked to evaluate the bactericidal and bacteriostatic activity. No differences were found in *B. cereus* and *P. fluorescens* compared with the previous check, recording an MBC of 20 µL and more than 40 µL, respectively; moreover in *E. coli* and *K. marina,* colonies were found in the 2 and 20 µL treatments while MBC was 40 µL. In *A. bohemicus*, the MBC was 20 µL.

## 3. Discussion

LEO liquid and vapour phases were investigated by HS-GC/MS analysis and the presence of monoterpenoids and sesquiterpenoids were highlighted. The most abundant components were linalool and linalyl acetate followed by eucalyptol and terpinen-4-ol, while lavandulyl acetate and borneol were identified as minor compounds. These molecules are mainly responsible for lavandin’s characteristic flavour and for their biological and therapeutic properties [29]. Depending on the different types of lavandin, variations in the chemical composition were found. Ghelardini et al. (1999) [30] described the chemical composition of *L. angustifolia* essential oil of Italian origin, characterised by linalool (31.5%), linalyl acetate (43.0%), and β-caryophyllene (5.0%) as the main compounds. Caputo et al. (2016) [31] analysed *L. angustifolia* essential oil obtained from the hydrodistillation of the plant grown in Salerno (Campania, Italy) and showed a composition rich in linalool (33.1%) and linalyl acetate (10.4%) and with low percentages of minor compounds. Iranian lavandin was characterised by 1,8-cineole (47.9%) as its main component followed by borneol (26.4%) and camphor (14.4%) [32] (Bajalan et al. 2017), while Romanian lavandin essential oil was richer in camphor and 1,8-cineole [33]. The essential lavender oil grown in India consisted of linalool (28.06%) and linalyl acetate (47.5%) [34]. The major compounds of a *Lavandula* hybrid grown in Northern Italy were linalyl acetate and borneol, which together accounted for 70% [35]. In our study, the sum of these two compounds did not exceed 25.8% and LEO revealed a high content of linalool (41.6%), while linalyl acetate reached 23.0%; 1,8-cineole (5.2%) and camphor (6.0%) were present as secondary compounds. A previous study carried out on the same type of lavandin grown in Tuscany, Italy, [36] reported that linalyl acetate amounted to 14.7%, 1,8-cineole 7.8%, and camphor 7.7%. All these variations in the percentages of the compounds detected could be either due to the cultivation area’s altitude or its microclimate. As for the quality of the LEO obtained, the component amounts found were in line with the levels accepted by the European Pharmacopeia (EPh) (Table 2). Only linalyl acetate was slightly below the limits set by EPh.

**Figure 1:**
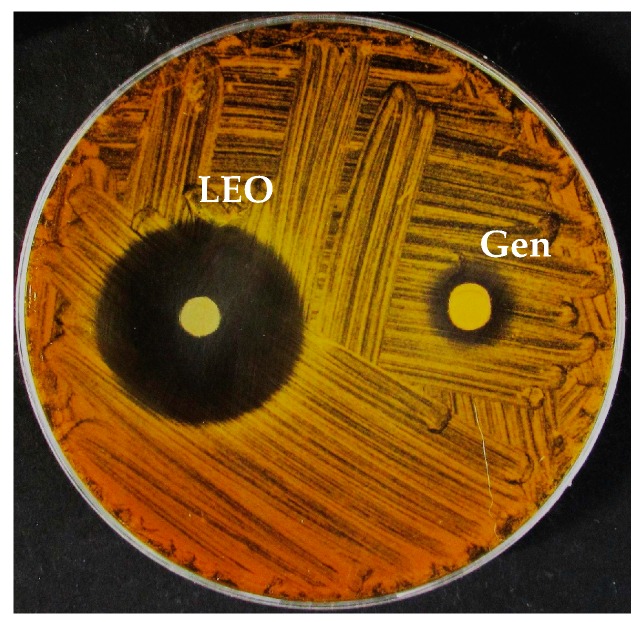
Representative image showing antibacterial activity against a bacterial strain.

The results obtained from the different tests on bacteria showed that *A. bohemicus* was the more sensitive strain to LEO with an inhibition halo of 47 mm and an MIC of 0.47% in the broth microdilution test, followed by *B. cereus* with a halo of 21.5 mm and an MIC of 0.94%. *E. coli* and *K. marina* showed similar values, both as regards the disk diffusion test (13.0 and 14.5 mm, respectively) and the microdilution test (both 1.87%). *P. fluorescens* proved to be the most resistant of all bacteria tested with an inhibition halo of 8.5 mm and an MIC of 3.75%. As seen in Table 4, the MBC values showed bactericidal activity of LEO at the MIC concentration for *A. bohemicus* and *E. coli*, while for *P. fluorescens* the MIC increased to 7.5%. This bactericidal effect occurred only for the G^−^ bacteria, while for the G^+^ bacteria bacteriostatic effects were observed, contributing to the formation of colonies after inhibition agent removal (results of the agar diffusion test are shown in Figure 1). Furthermore, in the VPT, *A. bohemicus* proved the most sensitive to LEO treatments with an MIC lower than the minimum concentration used for the test; *B. cereus*, *E. coli*, and *K. marina* recorded an identical MIC, varying slightly from the data previously obtained in the liquid phase. *P. fluorescens* achieved an MIC higher than the maximum concentration tested, 40 µL. 

## 4. Materials and Methods

### 4.1. Plant Materials

The LEO (IT BIO 007 D86K, lot number MGL01/18) was directly provided by the agricultural enterprise, Azienda Agricola Podere dell’Arco (Viterbo, Lazio), as a steam-distilled sample obtained from a cultivar of lavandin (*L. × intermedia* “Grosso”).

### 4.2. Gas Chromatography–Mass Spectrometry (GC–MS) Analysis

The essential oil was analysed by gas chromatograph directly coupled to a mass spectrometer (MS) Perkin Elmer Inc., Clarus 500 model (Waltham, MA, USA). The GC was equipped with two columns, one of which was a Restek Stabilwax (fused-silica) polar capillary column, and the other was a Varian (VF-1ms) apolar column. Helium was used as carrier gas at a flow rate of 1 mL/min [37]. The Restek column was operated using an injector temperature of 280 °C and the following oven temperature program: Isothermal at 60 °C for 5 min, then ramped to 220 °C at a rate of 5 °C min^−1^, and finally isothermal at 220 °C for 20 min. The other apolar column was operated using an injector temperature of 280 °C and the following oven temperature program: Isothermal at 60 °C for 5 min, then ramped to 220 °C at a rate of 5 °C min^−1^, a second ramp to 260 °C at a rate of 5 °C min^−1^ and finally isothermal at 260 °C for 10 min. One microlitre of lavandin EO was diluted in 1 mL of methanol and the injection volume was 1 μL. The injector split ratio was 1:20. Mass spectra were recorded at 70 eV (EI: electronic impact) and were scanned in the range 40–400 *m/z*. The GC-TIC (total ion current) mass spectra were obtained by data analysis software (TurboMass^TM^ GC/ MS PerkinElmer). The LEO constituents were identified by comparison of their linear retention indices (LRIs), (relative to C8–C30 aliphatic hydrocarbons, Ultrasci injected into both polar and apolar columns under the same operating conditions described above) with available retention data in the literature. Further identification of all components was made by matching their mass spectra with those stored in the Wiley and NIST02 mass spectra libraries database. GC-FID (flame-ionization detector) analysis was performed under the same experimental conditions using the polar column as described for the GC–MS measurements. The FID temperature was 280 °C. Relative amounts of the essential oil components were obtained by peak area normalisation without the use of an internal standard or correction factors and expressed in percentages. All analyses were repeated twice.

### 4.3. Headspace GC–MS Analysis

The volatile constituents were analysed by a PerkinElmer Inc., Headspace Turbomatrix 40 autosampler coupled to GC and GC–MS (Waltham, MA, USA). [38,39]. The procedures were optimised. The gas phase of the sealed vials was equilibrated for 20 min at 60 °C and was followed immediately by compound desorption into the GC injector in splitless mode. Quantification of compounds was performed by GC-FID under the same conditions described above.

### 4.4. Microorganisms Tested and Growth Conditions

Bacterial strains were obtained from the culture collections of Plant Cytology and Biotechnology Laboratory of Tuscia University and the Institute of Pharmacy and Molecular Biotechnology at Heidelberg University. Five microbial cultures belonging to gram-positive (G^+^) and gram-negative (G^−^) bacteria were tested: *E. coli* American Type Culture Collection-ATCC 25,922 (G^−^), *B. cereus* ATCC 10,876 (G^+^), *A. bohemicus* DSM 102,855 (G^−^), *K. marina* DSM 16,420 (G^+^), and *P. fluorescens* ATCC 13,525 (G^−^).

All tested bacterial strains were maintained on Lysogeny Broth agar (LB agar preparation: 10 g tryptone, 5 g yeast extract, 10 g NaCl, and 15 g agar per litre, autoclaved at 121 °C for 20 min) at different temperatures: 26 °C for *B. cereus, P. fluorescens*, and *A. bohemicus,* and 37 °C for *K. marina* and *E.* coli. All inocula were prepared with fresh cultures plated the day before the test.

### 4.5. Agar Diffusion Method

Fresh bacterial strains were suspended in Lysogeny Broth (LB preparation: 10 g tryptone, 5 g yeast extract, 10 g NaCl per litre, autoclaved at 121 °C for 20 min) to obtain a turbidity of 0.5 McFarland (approximately 10^8^ Colony Forming Unit/mL—CFU/mL) and then seeded on LB agar. Sterile disks (6 mm diameter) were placed on the agar and impregnated with 10 µL of pure LEO. As a positive control, 2 µL of gentamicin from a stock solution (10 mg/mL) was used.

The inhibitory activity was recorded as mm of halo diameter without growth around the disks [40]. Each bacterial strain was tested in duplicate and the halo of the inhibition zones measured using a vernier caliper rule. The average and the respective standard deviation (SD) of the three replicates were recorded.

### 4.6. Minimum Inhibitory Concentration (MIC)

The MIC is the lowest concentration of antimicrobial agent that completely inhibits growth of the organism in microdilution wells as detected by the unaided eye [40]. MIC values were determined according to the microwell dilution method [41], with small adjustments. Briefly, 96-microplate wells were prepared by adding 12 dilutions of LEO in Lysogeny broth, from 7.5% to 0.0037%, a growth control without treatments, a control with the same percentage (from 5% to 0.002%) of dimethyl sulfoxide (DMSO) used to solubilise LEO in an aqueous medium, a positive control with gentamicin diluted from 100 to 0.049 µg, and a sterility control without bacteria. In all wells, except for the sterility control, 50 µL of inoculum, previously diluted from 10^8^ to 10^6^ CFU/mL, were added and the plates were incubated for 20 h. To better visualise the inhibition activity, 20 µL of a solution 200 µg/mL of 3-(4,5-dimethylthiazol-2-yl)-2,5-diphenyltetrazolium bromide (MTT) were added. The assay was carried out in triplicate.

### 4.7. Minimum Bactericidal Concentration (MBC)

Before adding MTT to display the effective bacteria growth, 10 µL of the last four dilutions with no presence of turbidity were plated in an agarised Petri plate with LB agar and incubated for 24 h. The MBC was evaluated as the lowest concentration where no growth on agar occurred. The assay was carried out in triplicate.

### 4.8. Vapour Phase Test (VPT)

The antibacterial activity of LEO vapour phase was evaluated by the modified disk volatisation methods [42,43,44]. Briefly, 15 mL of liquid LB agar were poured into a 90 mm plastic Petri dish and 5 mL into its cover to prevent interactions between the plastic and LEO. After solidification, the fresh bacterial suspension containing 10^8^ CFU/mL was inoculated in the medium. Different aliquots of LEO were put in a 6 mm sterile disk: 40, 20, and 2 µL. A negative control was prepared with a plate without LEO. Liquid LB agar was put in the space between the cover and the base of the Petri dishes to facilitate the sealing. During the solidification time, the Petri dishes were sealed with the aid of an adhesive tape to hold the edges of the plate with its respective counterpart to prevent any LEO leakage. The dishes were put into a plastic bag and incubated for 24 h. After the incubation time, the presence or absence of microbes was visually evaluated and the plates were checked after seven days to investigate the bactericidal or bacteriostatic activity. All VPTs were carried out in triplicate.

### 4.9. Statistical Analysis

The results were expressed as means ± standard deviation (SD). Data were analysed with one-way analysis of variance (ANOVA) using GraphPad Prism software (GraphPad Prism 5.0, GraphPad Software, Inc., San Diego, CA, USA), with *p* values ≤ 0.05 as statistically significant.

## 5. Conclusions

This work aimed to chemically characterise the vapour and liquid phase of *L. × intermedia* “Grosso” essential oil (LEO).

Its main components were linalool and linalyl acetate followed by 1,8-cineole and terpinen-4-ol. Antimicrobial activity was also tested and the results showed a bactericidal effect on G^−^ bacteria and a bacteriostatic effect on G^+^ bacteria with an overlapping trend for the vapour and liquid phases. Lavandin, a natural sterile hybrid obtained by crossing *L. angustifolia* × *L. latifolia* and grown in the Lazio Region, Italy, was characterised for the first time in this work. Future studies will occur to test the single or combined activity of the main constituents and their possible uses in cosmetic, food, and pharmaceutical fields

## Figures and Tables

**Table 1:** Chemical composition (%) of *Lavandula × intermedia* cv. “Grosso” essential oil (LEO).

No.	Component ^1^	LRI^lit 2^	LRI ^3^	LRI ^4^	GC Peak Area (%)	HS-GC Peak Area (%)
1	α-pinene	1021	1019	935	1.1	8.7
2	camphene	1065	1055	946	0.5	3.7
3	β-pinene	1105	1100	981	0.4	2.8
4	myrcene	1156	1152	984	1.5	4.9
5	α-phellandrene	1160	1161	999	0.1	0.7
6	limonene	1198	1195	1025	1.0	3.5
7	1,8-cineole	1209	1210	1028	5.2	19.8
8	(*Z*)-β-ocimene	1237	1231	1038	0.9	-
9	γ-terpinene	1241	1235	1046	0.2	-
10	trans-β-ocimene	1276	1275	1055	0.7	-
11	terpinolene	1282	1283	1072	0.4	-
12	o-cymene	1287	1290	1025	0.2	1.7
13	hexyl isobutyrate	1335	1333	1145	0.3	-
14	1-octen-3-yl-acetate	1401.9	1400	1122	0.5	0.7
15	hexyl butanoate	1410	1414	1177	0.5	-
16	1-octen-3-ol	1458	1461	970	0.3	0.4
17	camphor	1507	1500	1133	6.0	4.4
18	linalool	1537	1539	1092	41.6	35.8
19	linalyl acetate	1553	1549	1245	23.0	7.5
20	lavandulyl acetate	1584	1580	1265	3.2	0.8
21	terpinen-4-ol	1603	1600	1174	4.8	2.9
22	(*E*)-β-caryophyllene	1613	1615	1428	1.3	-
23	(*Z*)-β-farnesene	1630	1635	1445	1.3	-
24	α-terpineol	1675	1680	1185	1.6	0.4
25	γ-muurolene	1685	1687	1508	0.5	-
26	borneol	1717	1721	1158	2.8	1.2
Total (%)					99.9	99.9
Monoterpenoids					89.2	94.4
Sesquiterpenoids					3.1	-
Others					7.6	5.5

^1^ Elution order on polar column; ^2^ Linear retention indices from literature; ^3^ Linear retention indices measured on polar column; ^4^ Linear retention indices measured on apolar column. HS-GC: Headspace coupled to gas chromatography.

**Table 2:** Percentage of the main compounds in the LEO sample in comparison with the accepted percentages by European Pharmacopoeia (EPh).

Component	EPh	Lavandin
linalool	25–45	41.6
linalyl acetate	25–46	23.0
1,8-cineole	max. 2.5	5.2
camphor	12–18	6.0

**Table 3:** Growth inhibition halo (mm) for LEO and the positive control (gentamicin) against the five tested Gram^+^ and Gram^−^ bacterial strains (*p* values ≤ 0.05).

	Agar Diffusion Test	LEO (10 µL)	Gen (20 µg)
	**Strain**	halo ± SD (mm)	halo ± SD (mm)
*Gram^−^*	*Escherichia coli*	13.0 ± 1.41	16.5 ± 2.12
	*Acinetobacter bohemicus*	47.0 ± 4.24	34.5 ± 0.70
	*Pseudomonas fluorescens*	8.5 ± 0.70	25.5 ± 0.70
*Gram^+^*	*Bacillus cereus*	21.5 ± 0.70	28.5 ± 0.70
	*Kocuria marina*	14.5 ± 2.12	27.0 ± 2.83

**Table 4:** Minimum inhibitory concentration (MIC) and Minimum bactericidal concentration (MBC) means for LEO expressed in percentage values (%) and the positive control (gentamicin) expressed in µg/mL against the five tested bacterial strains.

	Microdilution Test	LEO		Gen	
	Strain	MIC (%_Mean_)	MBC (%_Mean_)	MIC (µg/mL_Mean_)	MBC (µg/mL_Mean_)
*Gram^−^*	*E. coli*	1.87	1.87	3.12	6.25
	*A. bohemicus*	0.47	0.47	0.08	0.31
	*P. fluorescens*	3.75	7.51	1.56	6.25
*Gram^+^*	*B. cereus*	0.94	Bacteriostatic	1.56	3.12
	*K. marina*	1.87	Bacteriostatic	0.39	1.56

**Table 5:** LEO vapour phase test showing minimum inhibitory concentration (MIC) and minimum bactericidal concentration means (MBC) expressed in µL against the five bacteria tested.

	LEO VPT (Vapour Phase Test)		
	Strain	MIC (µL _Mean_)	* MBC (µL _Mean_)
*Gram^−^*	*E. coli*	20	40
	*A. bohemicus*	<2	20
	*P. fluorescens*	>40	>40
*Gram^+^*	*B. cereus*	20	20
	*K. marina*	20	40

* After seven days.
